# Cholinesterase Inhibitors in Mild Cognitive Impairment: A Systematic Review of Randomised Trials

**DOI:** 10.1371/journal.pmed.0040338

**Published:** 2007-11-27

**Authors:** Roberto Raschetti, Emiliano Albanese, Nicola Vanacore, Marina Maggini

**Affiliations:** 1 National Center for Epidemiology, Surveillance and Health Promotion, National Institute of Health, Rome, Italy; 2 Section of Epidemiology, Institute of Psychiatry, King's College London, London, United Kingdom; University of California Los Angeles Center on Aging, United States of America

## Abstract

**Background:**

Mild cognitive impairment (MCI) refers to a transitional zone between normal ageing and dementia. Despite the uncertainty regarding the definition of MCI as a clinical entity, clinical trials have been conducted in the attempt to study the role of cholinesterase inhibitors (ChEIs) currently approved for symptomatic treatment of mild to moderate Alzheimer disease (AD), in preventing progression from MCI to AD. The objective of this review is to assess the effects of ChEIs (donepezil, rivastigmine, and galantamine) in delaying the conversion from MCI to Alzheimer disease or dementia.

**Methods and Findings:**

The terms “donepezil”, “rivastigmine”, “galantamine”, and “mild cognitive impairment” and their variants, synonyms, and acronyms were used as search terms in four electronic databases (MEDLINE, EMBASE, Cochrane, PsycINFO) and three registers: the Cochrane Collaboration Trial Register, Current Controlled Trials, and ClinicalTrials.gov. Published and unpublished studies were included if they were randomized clinical trials published (or described) in English and conducted among persons who had received a diagnosis of MCI and/or abnormal memory function documented by a neuropsychological assessment. A standardized data extraction form was used. The reporting quality was assessed using the Jadad scale. Three published and five unpublished trials met the inclusion criteria (three on donepezil, two on rivastigmine, and three on galantamine). Enrolment criteria differed among the trials, so the study populations were not homogeneous. The duration of the trials ranged from 24 wk to 3 y. No significant differences emerged in the probability of conversion from MCI to AD or dementia between the treated groups and the placebo groups. The rate of conversion ranged from 13% (over 2 y) to 25% (over 3 y) among treated patients, and from 18% (over 2 y) to 28% (over 3 y) among those in the placebo groups. Only for two studies was it possible to derive point estimates of the relative risk of conversion: 0.85 (95% confidence interval 0.64–1.12), and 0.84 (0.57–1.25). Statistically significant differences emerged for three secondary end points. However, when adjusting for multiple comparisons, only one difference remained significant (i.e., the rate of atrophy in the whole brain).

**Conclusions:**

The use of ChEIs in MCI was not associated with any delay in the onset of AD or dementia. Moreover, the safety profile showed that the risks associated with ChEIs are not negligible. The uncertainty regarding MCI as a clinical entity raises the question as to the scientific validity of these trials.

## Abstract 2

A systematic review of trials of cholinesterase inhibitors for preventing transition of mild cognitive impairment (MCI) to dementia, conducted by Roberto Raschetti and colleagues, found no difference between treatment and control groups and concluded that uncertainty regarding the definition of MCI casts doubts on the validity of such trials.

## Editors' Summary

**Background.:**

Worldwide, more than 24 million people have dementia, a group of brain disorders characterized by an irreversible decline in memory, problem solving, communication, and other “cognitive” functions. The commonest form of dementia is Alzheimer disease (AD). The risk of developing AD increases with age—AD is rare in people younger than 65 but about half of people over 85 years old have it. The earliest symptom of AD is usually difficulty in remembering new information. As the disease progresses, patients may become confused and have problems expressing themselves. Their behavior and personality can also change. In advanced AD, patients need help with daily activities like dressing and eating, and eventually lose their ability to recognize relatives and to communicate. There is no cure for AD but a class of drugs called “cholinesterase inhibitors” can sometimes temporarily slow the worsening of symptoms. Three cholinesterase inhibitors—donepezil, rivastigmine, and galantamine—are currently approved for use in mild-to-moderate AD.

**Why Was This Study Done?:**

Some experts have questioned the efficacy of cholinesterase inhibitors in AD, but other experts and patient support groups have called for these drugs to be given to patients with a condition called mild cognitive impairment (MCI) as well as to those with mild AD. People with MCI have memory problems that are more severe than those normally seen in people of their age but no other symptoms of dementia. They are thought to have an increased risk of developing AD, but it is not known whether everyone with MCI eventually develops AD, and there is no standardized way to diagnose MCI. Despite these uncertainties, several clinical trials have investigated whether cholinesterase inhibitors prevent progression from MCI to AD. In this study, the researchers have assessed whether the results of these trials provide any evidence that cholinesterase inhibitors can prevent MCI progressing to AD.

**What Did the Researchers Do and Find?:**

The researchers conducted a systematic review of the medical literature to find trials that had addressed this issue, which met criteria that they had defined clearly in advance of their search. They identified three published and five unpublished randomized controlled trials (studies in which patients randomly receive the test drug or an inactive placebo) that investigated the effect of cholinesterase inhibitors on the progression of MCI. The researchers obtained the results of six of these trials—four examined the effect of cholinesterase inhibitors on the conversion of MCI to clinically diagnosed AD or dementia (the primary end point); all six examined the effect of the drugs on several secondary end points (for example, individual aspects of cognitive function). None of the drugs produced a statistically significant difference (a difference that is unlikely to have happened by chance) in the probability of progression from MCI to AD. The only statistically significant secondary end point after adjustment for multiple comparisons (when many outcomes are considered, false positive results can occur unless specific mathematical techniques are used to prevent this problem) was a decrease in the rate of brain shrinkage associated with galantamine treatment. More patients treated with cholinesterase inhibitors dropped out of trials because of adverse effects than patients given placebo. Finally, in the one trial that reported all causes of deaths, one participant who received placebo and six who received galantamine died.

**What Do These Findings Mean?:**

These findings suggest that the use of cholinesterase inhibitors is not associated with any delay in the onset of clinically diagnosed AD or dementia in people with MCI. They also show that the use of these drugs has no effect on most surrogate (substitute) indicators of AD but that the risks associated with their use are not negligible. However, because MCI has not yet been clearly defined as a clinical condition that precedes dementia, some (even many) of the patients enrolled into the trials that the researchers assessed may not actually have had MCI. Thus, further clinical trials are needed to clarify whether cholinesterase inhibitors can delay the progression of MCI to dementia, but these additional trials should not be done until the diagnosis of MCI has been standardized.

**Additional Information.:**

Please access these Web sites via the online version of this summary at http://dx.doi.org/10.1371/journal.pmed.0040338.

- An essay by Matthews and colleagues, in the October 2007 issue of *PLoS Medicine*, discusses how mild cognitive impairment is currently diagnosed
- The US Alzheimer's Association provides information about all aspects of Alzheimer disease, including fact sheets on treatments for Alzheimer disease and on mild cognitive impairment
- The UK Alzheimer's Society provides information for patients and caregivers on all aspects of dementia, including drug treatments and mild cognitive impairment
- The UK charity DIPEx provides short video clips of personal experiences of care givers of people with dementia

## Introduction

Alzheimer disease (AD) is a neurodegenerative disorder characterized by cognitive and memory deterioration, progressive impairment of activities of daily living, and a multiplicity of behavioural and psychological disturbances. AD is the main cause of dementia syndrome and one of the most burdensome conditions of later life. In a recent Delphi consensus study, based on a systematic review of the literature on the prevalence of dementia, the authors estimated that more than 24 million people worldwide currently have dementia [1]. Dementia is also the leading cause of disability in persons aged 60 y and older, and its direct and indirect costs are very high [2,3].

There exists little evidence of modifiable risk factors for AD [1]; disease-modifying therapies are not available [4], and of the symptomatic therapies the efficacy of cholinesterase inhibitors (ChEIs—donepezil, rivastigmine, and galantamine) in mild-to-moderate AD patients is questionable and has been widely debated [5,6].

In recent years, efforts have been made to study individuals believed to be at greater risk of developing dementia and who were considered as having mild cognitive impairment (MCI), which refers to a transitional zone between normal ageing and dementia [7–9]. However, widely accepted and validated criteria for diagnosing MCI do not exist, and the differences between this term and the other clinical labels given to the cognitive dysfunctions associated with aging are not clear (e.g., benign senescent forgetfulness, age-associated memory impairment, age-associated cognitive decline, mild cognitive decline, mild neurocognitive decline, and cognitive impairment no dementia) [10–14].

A number of longitudinal studies have attempted to estimate the rate of conversion from MCI to dementia. When comparing the different studies, the conversion rates vary greatly (from 9% to 40%) because of differences in sampling criteria, the case definition, the length of follow-up, assessment procedures, and the number of persons lost to follow-up. Moreover, up to 40% of MCI cases reverted to a normal cognitive condition within 2–3 y [15–18].

Despite the uncertainty regarding the definition of MCI as a clinical entity, clinical trials on ChEIs as a preventive treatment have been conducted in the attempt to study the possible role of these agents in slowing the onset of AD, because of the purported pathophysiological relationship between MCI and AD. The first published trials showed that these agents were not efficacious, yet the authors attributed these findings mainly to methodological issues, such as the selection of homogeneous samples, the definition of reliable outcomes, and the duration of treatment [19]. The substantial failure of these attempts was recently confirmed by two Cochrane systematic reviews, one on galantamine and the other on donepezil [20,21].

On the basis of the results obtained in the clinical trials on galantamine, in 2005 the US Food and Drug Administration [22] and health authorities all over the world issued a safety warning advising that galantamine should be used only for the approved indications of mild to moderate AD, and that for other possible indications (e.g., MCI) the risks may outweigh the benefit. Nevertheless, the suitability of using ChEIs to treat persons labelled with MCI continues to be widely debated [23–26].

Most of the trials conducted on the efficacy of donepezil, galantamine, and rivastigmine on MCI remain unpublished even after years from their conclusion. As a consequence, the original data are not available for researchers or physicians who could use these drugs in their clinical practice. In this context, we conducted a systematic review of published and unpublished trials on ChEIs, so as to provide an update on the risk–benefit profile for this drug class (donepezil, galantamine, and rivastigmine) in treating MCI.

## Methods

### Search Strategy

In February of 2006, we searched for the terms “donepezil”, “rivastigmine”, “galantamine” and “mild cognitive impairment”, and their variants, synonyms, and acronyms in the following sources: (1) four electronic databases—MEDLINE (http://www.pubmed.gov/; 1990 to February 2006), EMBASE (http://www.embase.com/; 1990 to February 2006), The Cochrane Collaboration (http://www.cochrane.org/index.htm), and PsycINFO (http://www.apa.org/psycinfo/); and (2) three registers—in particular, the Cochrane Collaboration Trial Register (http://www.thecochranelibrary.com/), Current Controlled Trials (http://www.controlled-trials.com/), and Clinicaltrials.gov (http://www.clinicaltrials.gov/). We also examined the bibliographies of all of the considered publications so as to identify other studies. We did not consider tacrine in our search, because it is no longer used in clinical practice, because of its high toxicity [27,28].

### Inclusion Criteria

Published and unpublished studies were included if they were: randomised controlled trials (RCTs) of cholinesterase inhibitors (donepezil, rivastigmine, galantamine); written in English; and conducted among persons with abnormal memory function documented by a neuropsychological assessment and/or who met diagnostic criteria for MCI.

All studies were required to have as an outcome measure the time to development of dementia or of possible or probable AD, or the improvement of measurement concerning cognitive/clinical/neuropsychiatric domains, and/or improvements based on neuroimaging examinations.

### Exclusion Criteria

Studies were excluded if the design was not an RCT; the study did not present original data; the study drug was not a ChEI; and participants were cognitively normal for age or had already been classified as having dementia of any type. Ongoing studies were not included in the review.

### Data Extraction

We screened the obtained titles, abstracts, and protocols. Data were extracted using a standardized data extraction form that was developed by all authors. The extracted information included doses of medication; duration of the trial; the number, age, and gender of participants; enrolment criteria; funding sources; primary and secondary outcomes; all-cause dropouts; adverse events; and deaths occurring during the study period. The data were extracted and summarized by two investigators (EA and NV) not blinded to the study's authors or to the publication status. To ensure that accurate data were obtained, a third investigator (RR) checked the extracted data, and discrepancies were resolved through discussions among all investigators.

### Data Analysis

When available, we recorded for each outcome the mean difference between baseline and follow-up measures for the individual study arms, and the standard deviation of each difference. When the standard deviation was not given it was estimated from the standard error. For each outcome the effect measure was estimated as the mean difference between treated and placebo groups. A Bonferroni correction for multiple comparisons was done for the individual tests within the same study.

We have not included any estimate of pooled effect because of clinical heterogeneity among the populations enrolled in the trials included in the review.

Statistical analyses were conducted by using Stata software 8.0 (Stata, College Station, Texas, United States).

### Assessment of Methodological Quality

To roughly measure the quality of the study design/reporting of each trial, we used the validated scale developed by Jadad and colleagues, which assigns a numerical score of 0–5 (5 being the best score) [29].

## Results

### Search Flow

**Figure 1:**
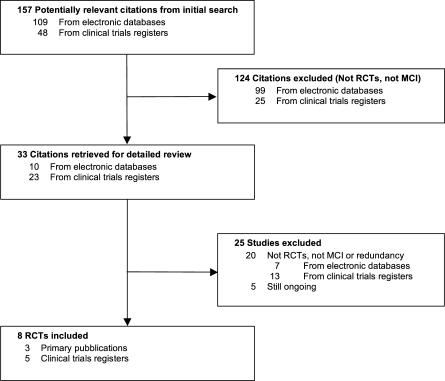
Trials Identification and Selection Process

The literature search yielded 157 potentially relevant citations: 109 studies from electronic databases and 48 from clinical trial registers. The selection process is illustrated in Figure 1. Of the 157 citations, 124 were excluded either because they were not RCTs (*n* = 119) or participants did not have MCI (five studies). After having evaluated the full text of the 33 remaining studies, we excluded 25 studies for the following reasons. Four were duplicates of other studies; nine had not been conducted among MCI patients (in most cases participants had already received a diagnosis of AD); six were not RCTs (three were comments or editorials on existing studies, and three were observational studies); one was conducted among persons not treated with ChEIs; and five trials were still ongoing.

### Characteristics of the Trials and Participants

The eight trials that investigated the efficacy and safety of ChEIs (three on donepezil, two on rivastigmine, and three on galantamine) in persons with MCI were included in the review (Table 1) [30–37]. Of these, three were published in peer-reviewed journals [34,36,37], and five were retrieved from clinical trial registers [30–33,35]. Regrettably, extensive synopses were available for only three of these five trials [31–33], whereas for the other two we were able to retrieve only a brief description of the principal characteristics [30,35]. Additional information were sought unsuccesfully from the original investigators and from the investigators' institutions. For this reason, these two trials are not always included when discussing the results. Notably, one of them was suspended, yet we were not able to obtain information from the manufacturers on the reasons for suspension [35].

**Table 1:**
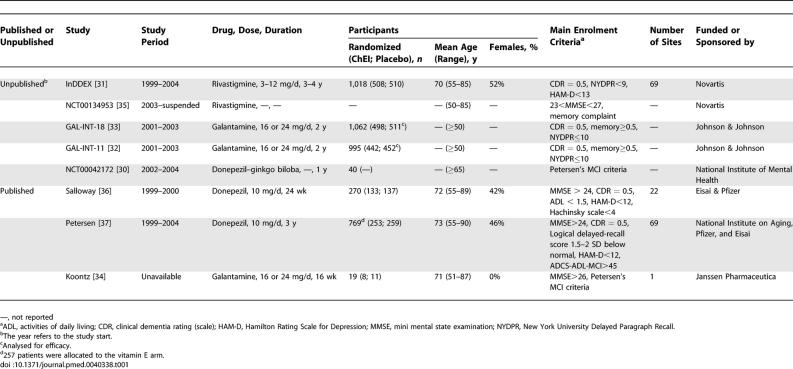
Characteristics of the Trials on Cholinesterase Inhibitors Included in the Review

The main characteristics of the eight trials are presented in Table 1. All trials but one were totally or partially sponsored by pharmaceutical companies [30]. The duration of the trials on donepezil was 24 wk, 1 y, and 3 y. Dosage was reported in two of these trials (10 mg/d after a starting dose of 5 mg/d) [36,37]. One of the three trials [34] on galantamine differed from the other two [32,33] in duration (16 wk versus 2 y), whereas dosages were identical in all three (16–24 mg/day). One of the two trials on rivastigmine lasted 3–4 y and applied dosages between 3 and 12 mg/d [31]; for the other trial, we were not able to retrieve information on either duration or dosage [35]. Of the six trials for which dosage was reported, in three, once the maximum dosage was reached, treatment continued with that dosage [34,36,37], whereas in the other three trials, the dosage remained flexible (16 or 24 mg for galantamine and 3–12 mg for rivastigmine) [31–33].

One trial was carried out in only one site [34]; the two published studies on donepezil were conducted in 22 and 69 centres, respectively [36,37]. One of the unpublished studies on rivastigmine was conducted in 69 centres. For the remaining four trials, this information was not available. The number of participants ranged from 19 to 1,062 and varied greatly among the trials. In all trials, all participants were greater than 50 y of age. When reported, the percentage of females ranged from 42% to 52%; in one trial, all 19 participants were males [34].

All studies enrolled patients accordingly to the Petersen's criteria: not demented, memory complaint, preserved general cognitive function, intact activities of daily living, impaired memory for age and education [9]. However, the operationalisation of the MCI diagnostic criteria differed widely among the trials (Table 1). Cognitive functions were assessed using the mini mental state examination (MMSE) with different cut-offs [34–35], or the clinical dementia rating (CDR) scale [31–33], or both [36,37]. Activities of daily living were assessed in two studies using two different scales (ADCL-ADL-MCI and ADL) [36,37]. The assessment of memory was based on the New York University paragraph recall test (immediate and delayed) in three studies [31–33], with two different cut-off scores, or on the Wechsler Memory Scale (Revised) Logical Memory delayed recall test [37]; three trials used two unspecified memory tests [32,33,35].

Only three of the trials [31,36,37] ascertained the psychiatric profile of participants, yet poorly, in that they only used the Hamilton Rating Scale for Depression, condition that may influence the execution of a neuropsychological test on memory. Finally, two studies [30,34] claim generically the use of Petersen's MCI criteria without any operational definition.

Information on the measurement tools used in the trials is provided in Text S1.

The use of this range of diagnostic criteria was an important source of clinical heterogeneity among the populations enrolled, except for two trials [32,33] that used essentially the same protocol.

### Study Quality

The quality assessment was carried out for all of the published trials [34,36,37] and for three of the unpublished ones [31–33]. However, it should be kept in mind that scarce or inadequate reporting does not necessarily imply that the methodology was of low quality.

The Jadad scores ranged from 2 to 3, indicating medium to low reporting quality ( Table 2 ). The description of the randomization process was adequate in only one trial [37]. In three trials, the description of this process was sufficient for understanding that the allocation of patients could not be predicted [32,33,37]. In all trials, the blinded status of the assessors, care providers, and participants was undermined by the fact that ChEIs have clear side effects. Three studies specified that the placebo was “indistinguishable” from the drug [32,33,37]. In one trial, an independent panel verified in double-blind conditions the occurrence of the primary outcome, yet not of all the surrogate measures [37]. The baseline characteristics of the participants were available only for the published studies. Four trials used the intention-to-treat (ITT) analysis [32,33,36,37]; however, only one carried out a sensitivity analysis [37].

**Table 2:**
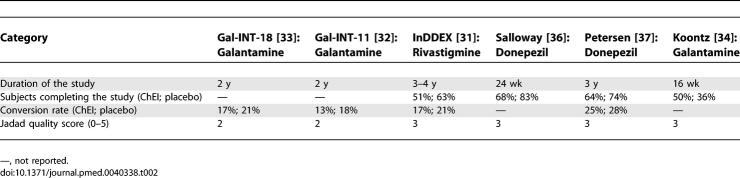
Conversion Rates from Mild Cognitive Impairment to Alzheimer Disease or Dementia and Jadad Quality Score

### Efficacy and Adverse Events

As mentioned above, we were able to obtain the results for six of the eight studies (Table 2). Conversion from MCI to AD or dementia was considered as the primary end point in four studies. In two studies, AD and dementia were diagnosed according to, respectively, NINCDS-ADRDA (National Institute of Neurological Disorders and Stroke–Alzheimer's Disease and Related Disorders Association) and DSM-IV (Diagnostic and Statistical Manual of Mental Disorders, 4th Edition) criteria [31,37], whereas in the other two, only a change in CDR score from 0.5 to 1.0 or higher was considered [32,33]. No significant differences emerged in the probability of conversion between the treated groups and the placebo groups. The rate of conversion ranged from 13% (over 2 y) to 25% (over 3 y) among treated patients, and from 18% (over 2 y) to 28% (over 3 y) among those in the placebo groups (Table 2). Only for two studies was it possible to derive point estimates of the relative risk of conversion: 0.85 (95% confidence interval 0.64–1.12) [31], and 0.84 (0.57–1.25) [37].

A reduced likelihood of conversion to AD, compared to the placebo group, was reported by one trial during the first 12 mo of treatment, yet this result was not observed at the end of the 3-y follow-up [37]. As discussed in the Cochrane review on donepezil, if we accept “an effect of donepezil in delaying AD for 12 months we must also accept that it then accelerates the appearance of AD after 18 months” [21].

The percentage of the study population that completed the study ranged from 51% to 68% among those allocated to treatment and from 36% to 83% among placebo recipients. The percentage of persons who completed the study was consistently lower for the treatment group, compared to the placebo group, except in the Koontz trial (Table 2). Furthermore, Petersen and colleagues reported that persons with more severe cognitive impairment were more likely to withdraw from the study and that there was a tendency for treated individuals to withdraw from the trial earlier [37]; thus the missing data were not randomly distributed. No study provided information that would allow the reader to assess the potential bias due to differential dropout (e.g., concomitant disorders).

The response to treatment was also assessed using a range of measures derived mainly from AD trials [19]. A total of 36 different scales, tests, and neuropsychological batteries, and two measures of volumetric imaging, were used as either a primary or a secondary end point (Table 3) (detailed information on the measurement tools used in the trials is provided in Text S1).

**Table 3:**
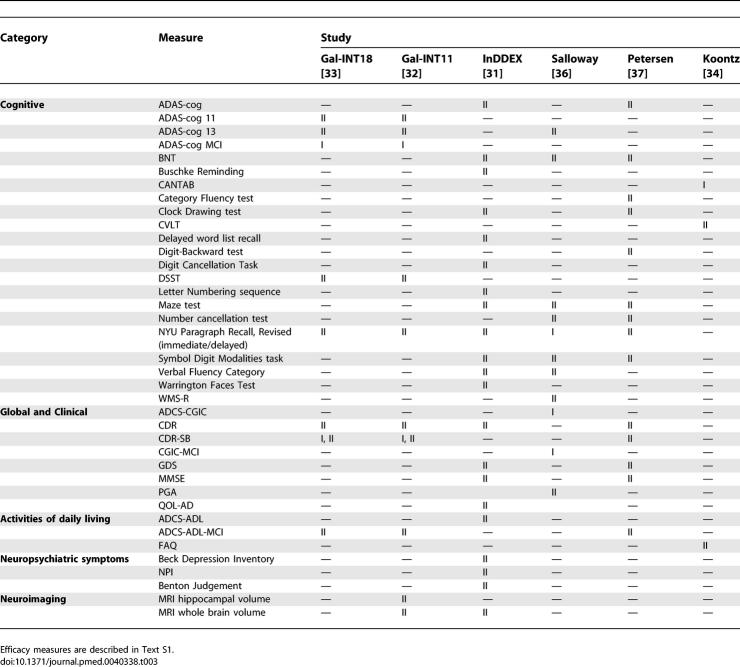
Primary (I) and Secondary (II) Efficacy Measures Used in the Trials Included

Efficacy was measured on an ITT population in four trials [32,33,36,37], and the last observation carried forward (LOCF) method was used for missing data points in three of them [32,33,36]. Only one trial adjusted the resulting *p*-values for multiple comparisons [37].

**Figure 2:**
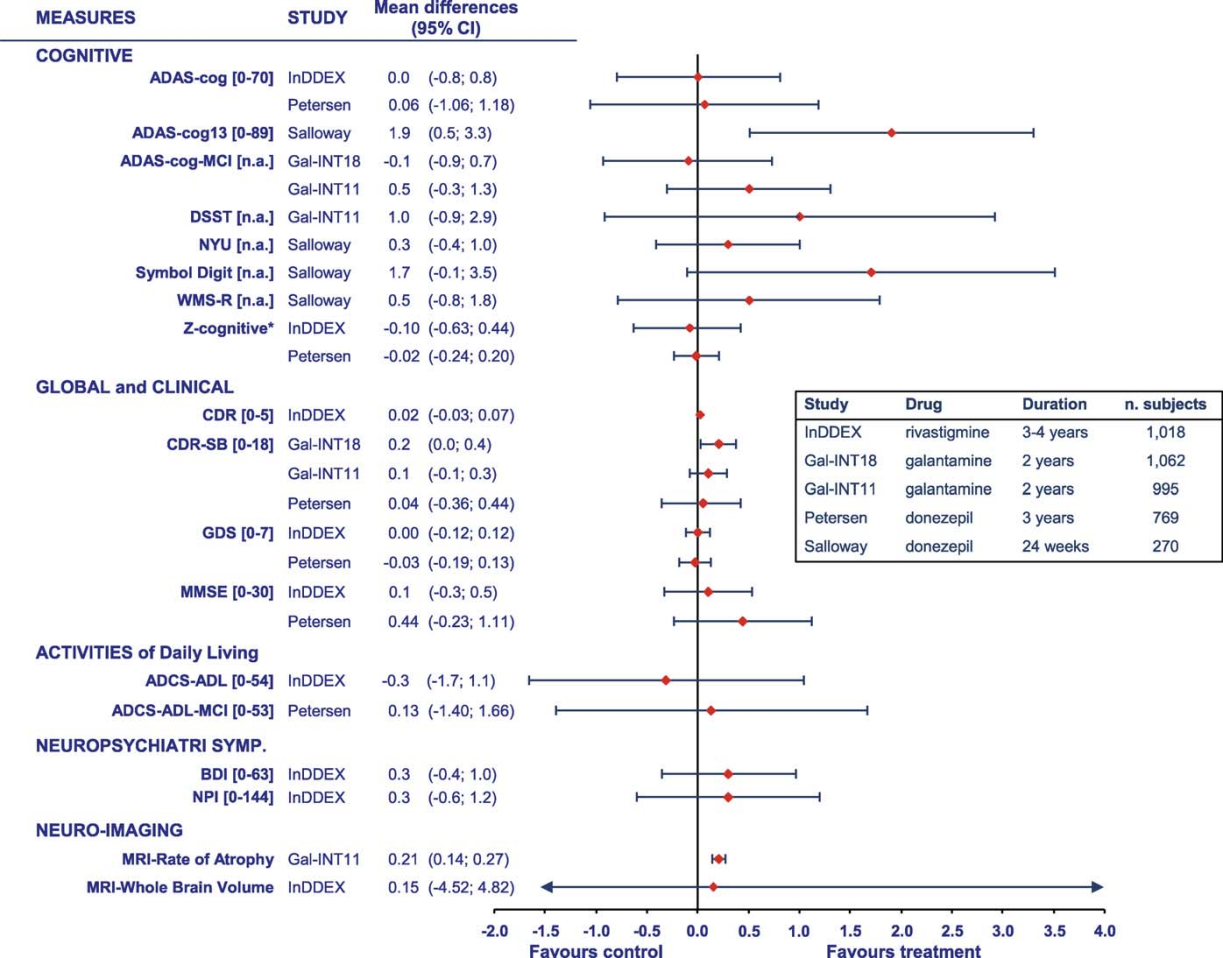
Effect of Treatment on the Efficacy Measures Used in the Included Studies Data points represent change in the treated groups versus the placebo groups. For each measure the range of possible scores are reported, when available, in square brackets. *Z-cognitive is a composite score based on a ten-test neuropsychological test battery in the InDDex study, and on a eight-test battery in the Petersen study.

In Figure 2 are reported the point estimates and confidence intervals for the outcomes for which specific results from the original studies were available. Data from one trial [34] were not considered as they actually refer to ten of the 19 trial participants, and they were not based on ITT analysis. Statistically significant differences emerged only for the mean rate of brain volume atrophy, and the CDR–Sum of the Boxes scores for galantamine [32]; and the cognitive functions evaluated by ADAS-cog 13 for donepezil [36].

When adjusting for multiple comparisons using the Bonferroni method, only the difference in the rate of atrophy in the whole brain remained significant.

The percentages of participants with at least one adverse event (AE), those with severe AEs, and those who discontinued for AEs were reported in only four trials [31–33,36]. Three trials described AEs occurring in at least 5% of participants [31,36,37]. One trial did not report AEs at all [34].

The percentage of participants with at least one AE was very high among both treatment recipients (88%–96%) and placebo recipients (73%–93%) (Table 4). The rate of discontinuation due to AEs was consistently higher for treatment recipients (21%–24%) than for placebo recipients (7%–13%). The data on causes of death were overall inadequate. Only GAL-INT-11 (the protocol for the galantamine RCT [32]) reported all of the causes of death for each study arm: one death occurred among placebo recipients (arrhythmia and cardiac arrest) and six deaths occurred among treatment recipients [32].

**Table 4:**
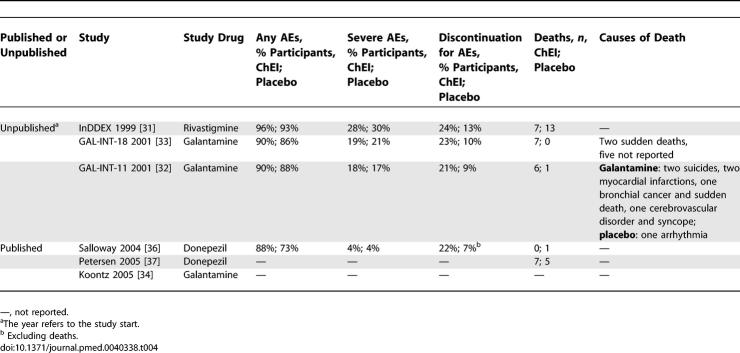
Summary of Data on Adverse Events Extracted from the Included Trials

## Discussion

The use of ChEIs in persons with MCI, for periods ranging from less than 4 mo to 3 y, was not associated with any delay in the onset of AD or dementia. Furthermore, according to the 38 surrogate measures used in the trials, and after appropriate adjustment for multiple comparisons, only neuroimaging showed a significant difference in favour of the drug being studied; the clinical implications of this finding are unclear [32]. Moreover, the safety profile showed that the risks associated with ChEIs are not negligible.

These results confirm for the all class of ChEIs those reported by two Cochrane systematic reviews. The first review was on the effect of galantamine in patient with MCI or AD; the authors concluded that the clinical benefit was marginal but “galantamine use in MCI is not recommended due to its association with an excess death rate” [20]. The second review [21] included two trials on donepezil, one that showed some promise for certain outcomes [36], and the other that showed side effects and no evidence of efficacy [37]. The authors' conclusion was “there is no evidence to support the use of donepezil for patient with MCI. The putative benefits are minor, short lived and associated with significant side effects” [21].

A recent meta-analysis on four trials using progression to dementia as the major parameter of efficacy found an approximate 24% reduction of risk of conversion to dementia and an increase of more than 50% in adverse events [38]. This pooled effect estimate was obtained notwithstanding the substantial heterogeneity of populations enrolled nor the methods of assessment. Moreover, the relative risk of conversion could not be calculated for the two trials on galantamine, as original data were necessary to apply a Cox proportional hazard ratio model.

Our revision of all the trials on the three drugs permits an overall comparison across the studies with respect to design, objective, and definition of MCI.

The primary end point of prevention trials should be the time to development of dementia or AD; this measure was used in only four of the trials included in this review [31–33,37]. The efficacy of the study drugs was also assessed on cognitive and/or functional domains applying a number of surrogate measures: 38 different instruments were used, considering simultaneously a wide range of hypotheses. Moreover, most of these measures have been developed for AD trials and transposed to MCI trials without first having been validated. However, it has already been claimed that the validation process is not simple, given that it is subordinate to the definition of MCI as a clinical entity, which itself is controversial [10–14,24].

A first important consequence of this uncertainty is that the trial populations were not homogeneous, even if the same criteria proposed by Petersen and colleagues were used [9]. Petersen and colleagues, in fact, did not specify which neuropsychological tests or instrument should be used to operazionalise MCI criteria. The predictable consequence of this flexibility is that operationalisation of the MCI diagnostic criteria differed widely among the trials, giving rise to quite different populations.

This was confirmed in a recent study in which the authors applied the enrolment criteria used in the GAL-INT-11, InDDEX, and Petersen et al. trial to the same cohort of 150 participants sampled in a memory clinic [15]. The study found that MCI was diagnosed in 51.3%, 21.3%, and 16.7% of the participants when applying, respectively, the criteria of GAL-INT-11, InDDEX, and Petersen's trial. This wide clinical heterogeneity among the study populations is the main reason for not combining the included studies in a pooled analysis.

In general, the uncertainty regarding MCI as a clinical entity raises the question as to the scientific validity and ethical value of these trials [39]. In fact, the requirement of scientific validity regards not only the mere technical domain of the correct design and conduct of a clinical trial. It is also a criterion to apply to the soundness of the clinical question approached by the experimentation. In a recent *New England Journal of Medicine* editorial, Karlawish used the concept of the “logic of clinical purpose” in discussing the role of clinical trials in AD [40]. According to this idea, ”Clinical trials are logically grounded in and ethically justified by the way they reflect and contribute to clinical practice.” As some participants in a 2006 workshop dedicated to MCI have reported, no agreement emerged during the conference as to the clinical utility of MCI, in that there was no consensus on the nature of MCI (is it a syndrome, a risk state, a new diagnosis?) [13].

Moreover, there is epidemiological evidence that the diagnosis of MCI is often unstable, and many persons labelled as having MCI may revert over time to normal cognition [16–18,23,24]. Since none of the trials provided information on this phenomenon, it cannot be excluded that some participants may have received the treatment despite having reverted to normal cognition.

A possible limitation of our review was that there was no information on the results of two trials: one of the trials was suspended for unknown reasons [35], and the other was completed in September 2004, yet the results are not yet available [30]. Nonetheless, this probably did not bias the risk–benefit profile of ChEIs for MCI patients, given that publication bias usually works in favour of the study drugs.

With regard to the quality of reporting of the trials, a common shortcoming was the inadequate description of the randomization and blinding procedures. Other important weaknesses were the very poor description of dropouts and of harm-related issues (e.g., only one trial reported all of the causes of death for each study arm). Our review was not blinded to the trials. This may have influenced our assessments of the quality of primary studies, although, given the simplicity of the Jadad scale, distorted judgements are very unlikely.

On average, the maximum dosage of ChEIs was used in all trials, which probably contributed to the high frequency of dropout and discontinuation for AEs. Mortality was higher among persons receiving donepezil or galantamine, compared to placebo recipients, and this excess in mortality seems to have been prevalently due to cardio- and cerebrovascular diseases. In GAL-INT-11 [32] and GAL-INT-18 [33], the regulatory authorities considered the increased mortality as noteworthy and stressed that ChEIs are not indicated for MCI patients; they also recommended that ChEIs be used with caution in AD patients with cardiovascular risk factors [22,41,42].

Recently, some authors have suggested the rating of medial temporal atrophy, performed using magnetic resonance imaging, as a routine clinical evaluation to identify “individuals with MCI who are destined to progress to dementia within 3 years” [43].

MCI seems to be an example of a risk factor conceptualised as a clinical condition, and it is surely still too heterogeneous and unpredictable as a clinical entity to enable researchers to establish its exact role in the progression toward dementia. From a public health point of view, it seems reasonable to affirm that additional research for clearly defining MCI is needed before testing new pharmacological treatments. When there is controversy surrounding the definition of a condition or disease, even inconclusive results from RCTs can be used to suggest treatment for persons tagged with some “pre-disease” condition. For example, in Italy an extimated 27% of patients diagnosed with MCI are prescribed cholinesterase inhibitors off-label [44]; it is likely that this situation is not limited to Italy.

The philosophy of widening the boundaries of treatable illness corresponds to the strategy of expanding the market for new products. This has been recently described as “disease mongering” [45,46]. This issue was also recently addressed by Britain's House of Commons Health Committee: “[…] There has been a trend towards categorising more and more individuals as ‘abnormal' or in need of drug treatment […]. Where disease awareness campaigns end and disease mongering begins is a very indistinct line” [47].

This review shows that the diagnosis of MCI is uncertain and variable, and whatever criteria are adopted, ChEIs are not effective in either preventing AD or improving cognitive functions in persons with MCI. These drugs may even be harmful in some people. Thus the alleged clinical implications of the trials, as claimed by some of the authors, are not justified by the data.

## Supporting Information

Text S1 Measurement Tools Used in the Trials (57 KB DOC)

## Abbreviations

AD: Alzheimer disease
AE: adverse event
CDR: clinical dementia rating
ChEI: cholinesterase inhibitor
MCI: mild cognitive impairment
MMSE: mini-mental state examination
RCT: randomized controlled trial
