# Supplementary material for: Cholinesterase Inhibitors in Mild Cognitive Impairment: A Systematic Review of Randomised Trials
Source: PLoS Med. 2007 Nov 27;4(11):e338. doi: 10.1371/journal.pmed.0040338 (PMC2082649; doi:10.1371/journal.pmed.0040338)
Supplement: Text S1 — (57 KB DOC) [file pmed.0040338.sd001.doc]

**APPENDIX**

**COGNITIVE MEASURES**

**ADAS-cog (/11; /13; MCI)**: Alzheimer’s disease assessment scale-cognitive subscale (11 and 13 subitems; Mild Cognitive Impairment); the original ADAS-cog is a psychometric scale consisting of 11 items that evaluate selected aspects of memory, orientation, attention, language, reasoning and carrying out instructions, it ranges from 0 to 70 and for all its variants higher scores indicate poorer function

**BNT**: Boston Naming Test; that assesses the ability to name pictures of objects through spontaneous responses and need for various types of cueing

**Buschke Reminding**: a test to measure verbal learning and memory during a multiple-trial list-learning task

**CANTAB**: Cambridge Automated Neuropsychiatric Test Assessment Battery; including: Cambridge Gambling Task, Affective Go/No-go, Verbal Recognition Memory, Graded Naming Test, Simple Reaction Time and Choice Reaction Time for a total of nineteen computer-based tests grouped in broad functional categories

**Category Fluency test**: quickly producing words in a specified category (e.g. animals, fruits, vegetables) is the major ability required by this test

**Clock Drawing test**: a test for visuospatial skills

**CVLT**: California Verbal Learning Test; a immediate free recall which examines several aspects of verbal learning, organization, and memory

**Delayed word list recall:** a test of memory

**Digit-Backward test:** a test to test executive function

**Digit Cancellation Task:** this task is essentially a measure of short-term memory and reaction time

**DSST**: Digit Symbol Substitution Test; an attention performances measure

**Letter Numbering sequence:** a subtest of the Wechsler Adult Intelligence Scale that assess the performance to read a sequence of numbers and letters and to recall the numbers in ascending order and the letters in alphabetical order

**Maze test** : a nonverbal test of performance intelligence consisting in a graded set of paper forms on which the subject traces the way from a starting point to an exit

**Number Cancellation test**: a test which mainly assess attention and reasoning

**NYU PR**: New York University Paragraph Recall; a test of memory

**Symbol Digit Modalities Task**: a test to test executive function

**Verbal Fluency** **category**: a test designed to measure the speed and flexibility of verbal thought processes

**Warrington Faces Test:** a widely used instrument for detecting impairment in visual memory

**WMS-R**: Wechsler Memory Scale-Revised; a test that yield information about various kinds of memory and learning processes and provide a comprehensive assessment of memory

**GLOBAL AND CLINICAL MEASURES**

**ADCS CGIC**: Alzheimer’s disease Cooperative Study’s Clinical global impression of change (for MCI); a clinical global impression of change scale for use with patients with aMCI based on semistructured interviews from both the subject and the informant

**CDR**: Clinical Dementia Rating (scale); The Clinical Dementia Rating Scale (CDR), a comprehensive structured interviews based on worksheets. CDR assesses dementia severity by staging, and includes cognitive, functional and social domains in the overall staging

**CDR-SB**: Clinical Dementia Rating–Sum of the Boxes; Its variant, the CDR sum of the boxes (CDR-SB), is a more quantitative numerical rating (it merely sums up the scores of the six individual domains of the CDR)

**CGIC-MCI:** a clinical global impression of change scale for use with patients with aMCI based on semistructured interviews from both the subject and the informant

**GDS**: Global Deterioration Scale; the Global Deterioration Scale (GDS) which rates seven stages of dementia, with higher scores rating poorer cognition, assessing the phenomenologic global progression of AD cognitively, functionally and behaviourally without structured interview

**MMSE**: Mini Mental State Examination; The MMSE is a brief, quantitative measure of cognitive status in adults

**PGA**: Patient Global Assessment; a measure of global function

**QOL-AD**: Quality of Life Alzheimer’s Disease; an questionnaire on perceived quality of life in AD patients as rated by patients and caregivers

**ACTIVITIES OF DAILY LIVING**

**ADCS-ADL**: Alzheimer’s disease Cooperative Study Activities Living

**ADCS-ADL-MCI**: Alzheimer’s disease Cooperative Study Activities Living adapted to MCI

**FAQ**: Functional Activities Questionnaire. The FAQ is an informant-based measure of functional abilities. Informants (i.e., family members, caregivers) rate the patient's performance of 10 complex, higher order activities. The higher the score (max 30), the poorer the function

**NEUROPSYCHIATRIC SYMPTOMS**

**Beck Depression Inventory**: a test to assess the depression

**HAM-D**: Hamilton Rating Scale for Depression

**NPI**: Neuropsychiatric Inventory; a battery including a standardised assessment of neuropsychiatric symptoms

**Benton judgment of line**: a test to assess the characteristics and the anomalies of the perception

**NEUROIMAGING MEASURES**

**MRI**: Magnetic Resonance Imaging
